# Improving Depth Estimation by Embedding Semantic Segmentation: A Hybrid CNN Model

**DOI:** 10.3390/s22041669

**Published:** 2022-02-21

**Authors:** José E. Valdez-Rodríguez, Hiram Calvo, Edgardo Felipe-Riverón, Marco A. Moreno-Armendáriz

**Affiliations:** Centro de Investigación en Computación, Instituto Politécnico Nacional, Av. Juan de Dios Bátiz s/n, Ciudad de México 07738, Mexico; gorillazclint@hotmail.com (J.E.V.-R.); hcalvo@cic.ipn.mx (H.C.); edgardo@cic.ipn.mx (E.F.-R.)

**Keywords:** depth estimation, hybrid convolutional neural networks, semantic segmentation, 3D CNN

## Abstract

Single image depth estimation works fail to separate foreground elements because they can easily be confounded with the background. To alleviate this problem, we propose the use of a semantic segmentation procedure that adds information to a depth estimator, in this case, a 3D Convolutional Neural Network (CNN)—segmentation is coded as one-hot planes representing categories of objects. We explore 2D and 3D models. Particularly, we propose a hybrid 2D–3D CNN architecture capable of obtaining semantic segmentation and depth estimation at the same time. We tested our procedure on the SYNTHIA-AL dataset and obtained $\sigma_{3}=0.95$, which is an improvement of 0.14 points (compared with the state of the art of $\sigma_{3}=0.81$) by using manual segmentation, and $\sigma_{3}=0.89$ using automatic semantic segmentation, proving that depth estimation is improved when the shape and position of objects in a scene are known.

## 1. Introduction

Depth estimation from a single image consists of calculating the distance between the objects in an image to the user’s point of view. This distance is calculated through a pair of images obtained from both eyes (binocular vision) by using the overlap between the field of view of both eyes [1]. Depth estimation from a single image is a complex task since a single or monocular image can have a greater number of depth signals, such as perspective, interposition, lighting, focusing, etc. [2], and unlike depth estimation from binocular images, most of the cases only use the disparity to calculate depth. An ideal system would analyze all these signals to obtain a better depth representation from the image. With the help of deep neural networks, we believe that it is possible to get most of these signals and perform depth estimation. A common problem of this approach is that despite neural networks being capable of extracting all the necessary information from a single image, they tend to ignore small objects on the image, or sometimes these objects are fused with the background [3]. In this work, we propose to improve the network’s ability to identify individual objects with local information such as that obtained from semantic segmentation. Our algorithm first identifies pixels contained in an image as meaningful classes of objects; these classes are semantically interpretable and correspond to real-world categories. Once we identify the objects in the image, we proceed to estimate depth using this information. To carry out these objectives, we propose the use of 2D and 3D Convolutional Neural Networks (CNN) trained with a synthetic dataset, containing both semantic segmentation and depth information, as well to explore a hybrid 2D–3D CNN model capable of estimating depth from a single image, while at the same time, segment objects found in it.

This work is divided as follows: Section 2 describes the state of the art and related works. Section 3 describes the proposed methodology, Section 4 describes the experiments and the results obtained in this work, and finally, in Section 5, we draw our conclusions.

## 2. Related Works

Depth estimation from a single image using CNNs has been studied in recent years; the first work that uses CNNs for depth estimation was proposed by Eigen et al. [4]. They proposed two CNN models that estimate depth from a single RGB image: The first CNN estimates global depth and the second CNN refines the local view of the first CNN. Eigen and Fergus [5] propose three CNNs: The first network estimates depth at a global view, the second network tries to estimate the depth at half the resolution of the input image, and a third one refines or estimates depth at a local level; in both works, they use a modified scale-invariant mean squared error as loss function. Liu et al. [6] use a CNN combined with Conditional Random Fields (CRFs). The CNN first extracts depth at the global level and the CRFs refine the obtained depth, Mousavian et al. [7] pursue a similar purpose using a CNN model to extract features and CRFs to classify depth values. Afifi and Hellwich [8] propose a single CNN used to estimate depth with their loss function. Laina et al. [9] use a fully convolutional CNN with upsampling embedded. Li et al. [10] use dilated convolutions on their CNN and soft-weight-sum inference. Xu et al. [11] use a CNN with multiple CRFs. Finally, Koch et al. [12] make an analysis and comparisons between all the methods mentioned before. Atapour-Abarghouei and Breckon [13] use an arrangement of eight CNN models (U-Net) [14] that first estimates the semantic segmentation of the image and then estimates depth from the segmented objects. Lin et al. [15] proposed an architecture that joins a CNN that estimates depth and separately, a CNN that estimates the semantic segmentation. Yue et al. [16] use two CNN models: the first one estimates depth from the RGB image and the second one estimates it from the semantic segmentation. Sun et al. [17] used an encoder-decoder CNN that estimates semantic segmentation; this encoder-decoder CNN internally performs depth estimation to improve semantic segmentation. Wang et al. [18] use a deep CNN to obtain depth information and the 2D location of certain objects in the image; they use the bounding box methodology to obtain the location of the objects instead of the semantic segmentation. Genovese et al. [19] propose encoder-decoder CNN models built from ResNet50 [20] and PSPNet [21], to obtain both semantic segmentation and depth estimation separately.

In recent works, authors have shown that depth information and semantic segmentation go hand in hand since using both can improve one of them. In the same way, CNN architectures have been used both as sources of information, however they use 2D CNN architectures in which the two-dimensional operations of the same convolution operation somehow flatten the input in which some features may be lost. Therefore, in this work, we propose the use of 3D CNNs, in which we create 3D volumes of data and extract features from them to estimate depth. In the same way, we will use a 2D CNN to estimate the semantic segmentation and finally create a hybrid CNN to estimate the depth with embedded semantic segmentation.

## 3. Proposed Methodology

In this work we propose a new methodology consisting of combining local information (i.e., the objects of the image) with global information (the background of the image); in other words, by knowing the position and shape of the objects, we expect to improve detecting the depth in which these are found in the image, as mentioned in Howard [2]. First, we give a brief description of the semantic segmentation and the automatic extraction of the objects; then we focus on the estimation of the depth using CNNs from a single image and its semantic segmentation added as additional input channels; finally, we build an architecture capable of simultaneously estimating depth and the semantic segmentation of a single RGB image.

### 3.1. Semantic Segmentation

**Figure 1:**
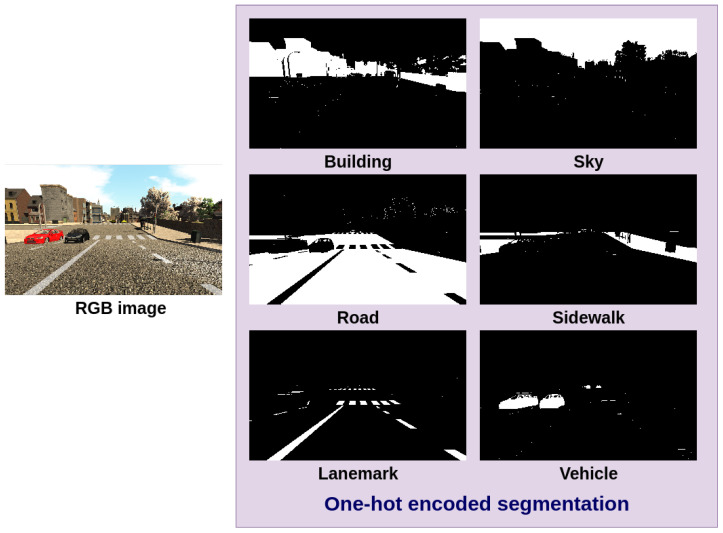
One-Hot Encoded Semantic Segmentation (OHESS) from a RGB image (not all classes are included in the figure).

The semantic segmentation consists of the classification of pixels from an image into meaningful classes of objects; this segmentation is represented in a One-Hot Encoded Semantic Segmentation (OHESS), in which each one of the classes is represented as a single binary image (plane) as shown in Figure 1, in which a white pixel represents the presence of an object of a certain class.

**Figure 2:**
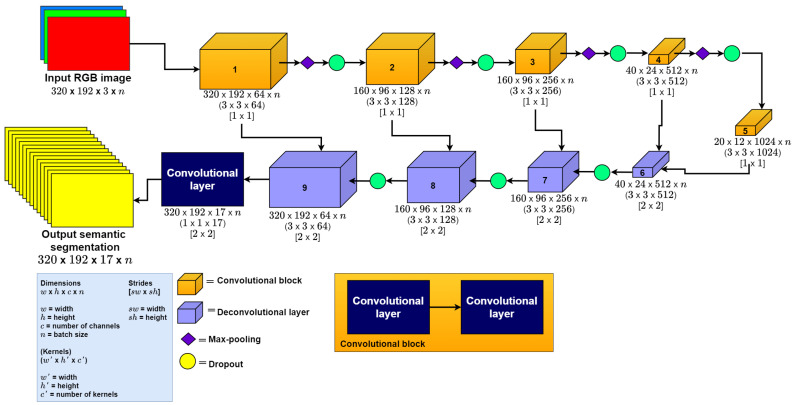
U-Net implementation.

**U-Net CNN.** To automatically obtain the semantic segmentation for the final model described in Section 3.3, we used the original U-Net CNN model proposed by Ronnenberg [14]. This is a 2D CNN with layers ordered as an auto-encoder architecture. We selected this model because it has shown good results in previous semantic segmentation works [22,23]. Additionally, it can also be adapted to any input and output size. Finally, we chose the U-Net because it is easy to implement, and training time is lower than other state-of-the-art models [14]. This model receives as input an RGB image and the output of the model is the One-Hot Encoded Semantic Segmentation. We modified the input dimension of the original U-Net model from 512 × 512 × 1 to 320 × 192 × 3 (the last dimension corresponds to the number of channels in the RGB image) and the output from 512 × 512 × 2 to 320 × 192 × 14 (the last dimension corresponds to the number of classes in the semantic segmentation). We implemented this model as depicted in Figure 2, adapting the input and output size of the model; all the layers in this model use ReLU as the activation function, except the last layer, which uses Sigmoid as the activation function.

### 3.2. Depth Estimation Architectures

**Figure 3:**
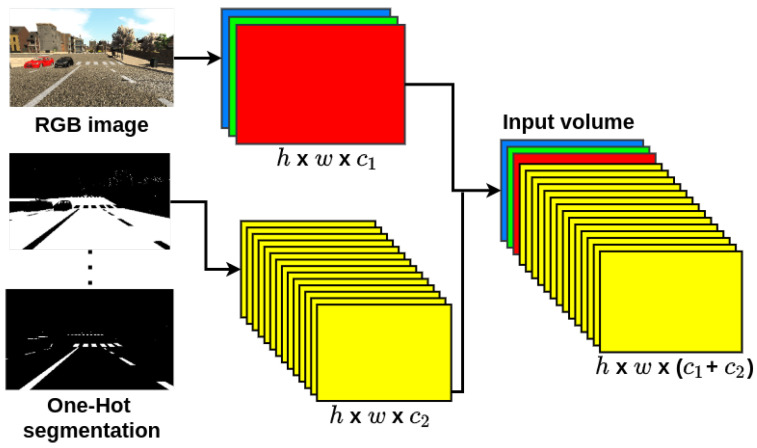
Input volume.

To estimate depth from an RGB image and its semantic segmentation we propose the use of 2D and 3D CNNs. Firstly, the input volume for all the proposed CNN models consists in a concatenation between the RGB image and its One-Hot Encoded Semantic Segmentation (OHESS) as depicted in Figure 3; by doing this we feed both signals into the CNNs as a single volume.

**Figure 4:**
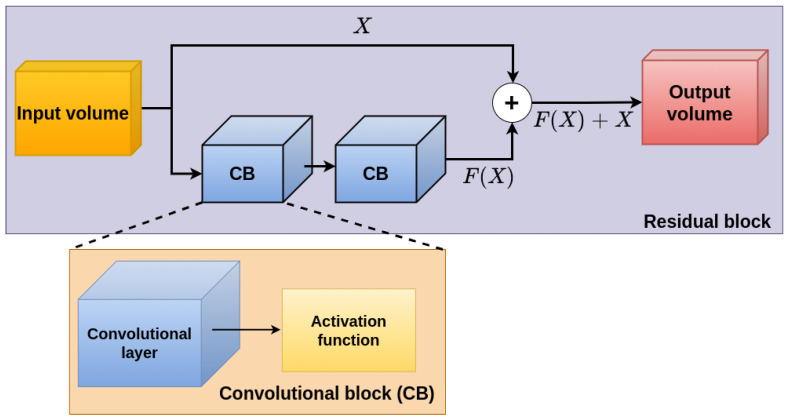
Residual block implementation.

As a first approach, we use a 2D CNN proposed by Valdez et al. [24], in the reSidual-Convolutional-Refinement (SCRX) CNN model. This model is composed of two stages: The first one is the feature extraction stage, which extracts the features from the input volume, consisting of four Residual Blocks [20] (see the implementation of a residual block in Figure 4) with the Rectified Linear Unit (ReLU) [25] as the activation function; we used the residual block to avoid weights with a zero value and kernel size of 3 × 3 in these blocks. Max-pooling is used with a kernel size of 2 × 2, only on the first two Residual Blocks to reduce image resolution and reconstruct depth at different image sizes. The output of this stage consists of a convolutional layer [26] with a sigmoid activation function, to limit the output to values between 0 and 1. This final layer performs depth estimation globally.

The second stage is the Refinement stage, which extracts additional information from the input, consisting of two convolutional blocks with a kernel size of 3 × 3 and ReLU as the activation function and both layers followed by max-pooling layers [27] with a kernel size of 2 × 2. This stage was based on the method proposed by Xu et al. [28]. This method consists of extracting additional features from the input images at two different sizes and joining them with the output layer.

**Figure 5:**
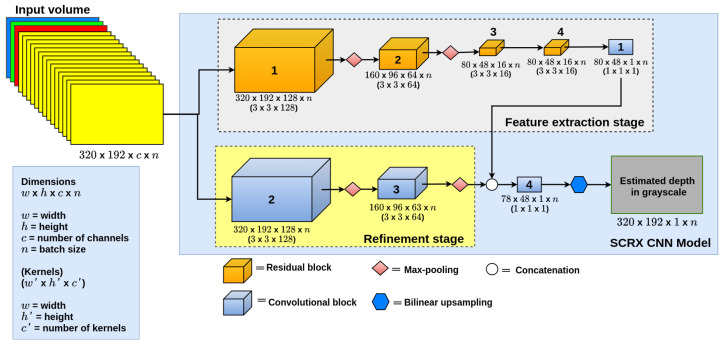
SCRX CNN model.

The output of the model is given by a convolutional layer with kernel size 3 × 3 and a sigmoid activation function followed by a bilinear upsampling layer [14], used to retrieve the size of the original image. Although the SCRX model appears to be a multi-stage model, it is single stage since, once the model is assembled, it is fully trained with the whole train dataset, unlike the models proposed by Eigen and Fergus [5], which train each of the stages separately. For example, if our model were multi-stage, the refinement stage is trained with the complete dataset; once it is trained, it would be added to the full model. This model is depicted in Figure 5.

**Figure 6:**
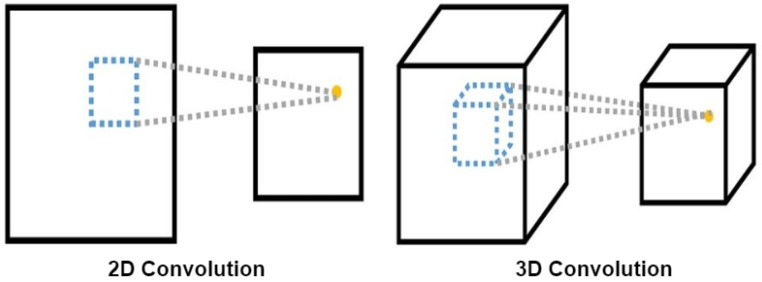
2D vs. 3D convolution.

**3D CNN models.** To extract more features from the semantic segmentation and the RGB image together, we opted to explore the use of 3D CNNs, since this type of convolution extracts and processes 3D volumes of information. Another important difference between 2D and 3D convolution is the way of processing the images, since in our case the 2D network extracts the characteristics of each of the input images separately. 3D convolution, on the other hand, extracts characteristics from grouped planes as can be seen in Figure 6. In this work, we propose two 3D CNN architectures: 3D CNN-S and 3D CNN-UP.

**Figure 7:**
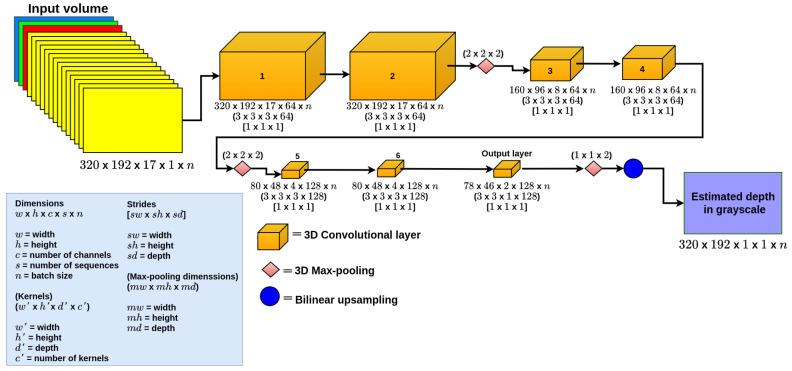
3D CNN-S.

**3D CNN-S.** This is a simple 3D CNN model based on LeNet [29], with which we explore the capabilities of the 3D CNN. It is composed of seven 3D convolutional layers as depicted in Figure 7, in which the six first layers extract features from the input and the last layer is the output of the model. This last layer is composed of a 3D convolutional layer followed by a max-pooling layer. To recover the size of the input image, we added bilinear upsampling after the output. All the convolutional layers use ReLU as an activation function, except the last layer, which uses a sigmoid activation function.

**Figure 8:**
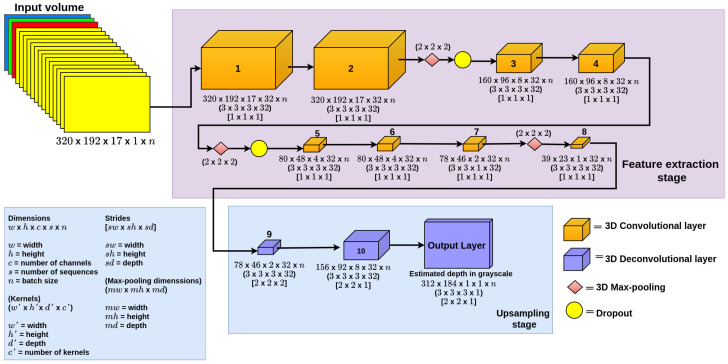
3D CNN-UP.

**3D CNN-UP.** This proposed model is based on the U-Net as it is capable of recovering the original size of the input image. This model is composed of two stages: The feature extraction stage, which is made of eight 3D convolutional layers. This stage is capable of extracting features from the input and reducing them into a smaller representation. The upsampling stage is composed of three 3D deconvolutional layers that try to estimate the depth and recover the size of the original image. The output of the model is made by a 3D deconvolutional layer, in which the resulting estimated depth map is given as a grayscale image. In this model, all the convolutional and deconvolutional layers use ReLU as an activation function. The block diagram of this model is shown in Figure 8. Optionally, we added a dropout equal to 0.5, to avoid overfitting (3D CNN-UP${}^{do}$).

### 3.3. SSegDep-Net: Hybrid 2D–3D CNN Architecture

**Figure 9:**
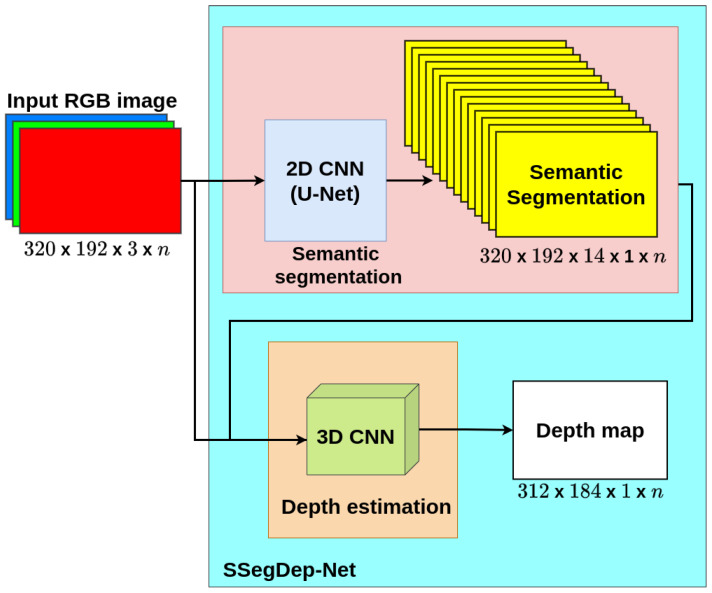
SSegDep-Net model.

In this work, we propose a hybrid 2D–3D CNN capable of estimating at the same time the semantic segmentation and its depth from a single RGB image: The **SSegDep-Net**. It is mainly composed of two modules, the segmentation and depth estimation modules. The semantic segmentation module consists of a 2D CNN capable of estimating the semantic segmentation from an RGB image based on the U-Net network described in Section 3.1. The depth estimation module consists of a 3D CNN that receives as input the output of the semantic segmentation module and the RGB image to obtain an estimation of depth from these data. Each module is trained separately; once trained, the SSegDep-Net is ready to estimate depth from a single RGB image. This model is shown in Figure 9.

## 4. Experiments and Results

This section describes the results and some details related to the training in the proposed models. First, we describe the dataset used to perform our experiments. Secondly, we describe some implementation details of the models. Then we show the results of the semantic segmentation algorithm and the results of the depth estimation architectures. Finally, we show the results of the SSegDep-Net and the evaluation of all the experiments proposed in this work.

### 4.1. Dataset

The proposed method is a supervised algorithm; therefore, we used the SYNTHIA-AL dataset [30] for training and testing. This dataset contains images of a virtual world, specifically urban scenes and additional information, such as semantic segmentation given as an image with pixel values between 0 and 13, representing the label *l* and depth information coded as a grayscale image with depth values *d* between 0–255. The SYNTHIA-AL dataset is divided into training and testing subsets. The training dataset consists of approximately 198,000 RGB images, including their semantic segmentation and depth information, and the testing set contains approximately 40,000 images, including their semantic segmentation and depth information as well. For our experiments, we will use the segmentation information present in the training subset for the SCRX and all 3D CNN Models: 3D CNN-S and 3D CNN-UP. The SSegDep-Net model will not use this information, as it will estimate it from the trained U-Net described in Section 3.1.

We decided to use a synthetic dataset because labels and depth estimation are automatically and precisely generated, while datasets based on real images, such as KITTI [31] and Cityscapes [32], have depth estimation estimated by LiDaR scans. These scans provide a sparse depth map and, apart from being inaccurate, they need to be converted to a depth map to use in CNNs, which is possible, however it adds a layer of possible inaccuracies. Additionally, although both datasets have manually labeled semantic segmentation, they do not provide both semantic segmentation and depth estimation in the same dataset.

### 4.2. U-Net Semantic Segmentation Results

**Figure 10:**
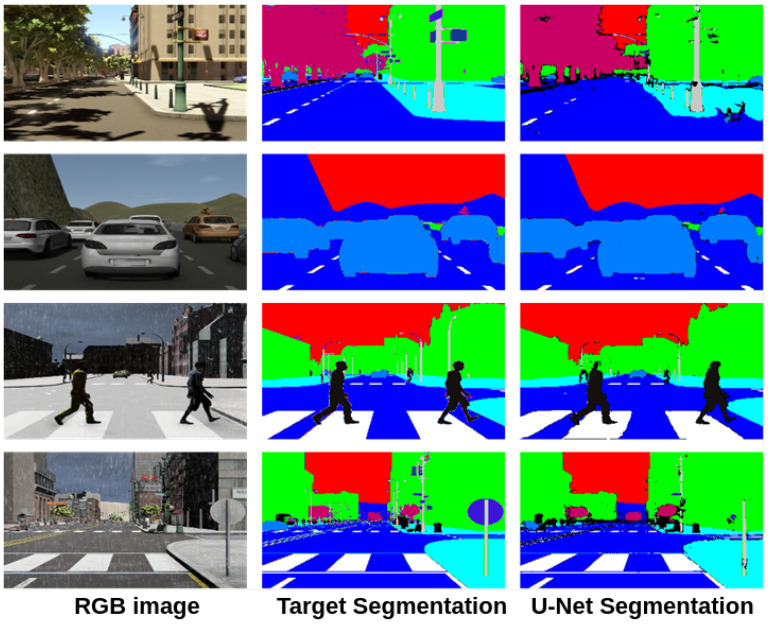
Results obtained with the U-Net model.

As mentioned in previous sections, we will use the U-Net model to automatically obtain the semantic segmentation; the One-Hot Encoded Semantic Segmentation (OHESS) consists of 14 binary planes (see Figure 1), corresponding to the total number of classes or labels (14 labels: Miscellaneous, Sky, Building, Road, Sidewalk, Fence, Vegetation, Pole, Vehicle, Sign, Pedestrian, Cyclist, Landmark, and Traffic light). This model was trained using the given semantic segmentation and the RGB image by the SYNTHIA-AL dataset. In Figure 10, some qualitative results are shown in a color map representation in which each color represents a single class.

We used the binary cross-entropy function as the loss function for the U-Net, given by Equation (1), where $y^{\prime }$ is the estimated segmentation, *y* is the target segmentation, and *N* is the total number of pixels in the image:

(1) $$\left[H\right]\mathrm{Loss}=-\frac{1}{N}\sum_{i=1}^{N}y_{i}\cdot \log y_{i}^{\prime }+\left(1-y_{i}\right)\cdot \log\left(1-y_{i}^{\prime }\right).$$

To evaluate quantitatively the performance of U-Net, we used the Intersection over Union (*IoU*) metric, described in Equation (2); this is a number from 0 to 1 that specifies the number of overlapping pixels between the predicted and target segmentation. In Table 1, we show the results of the *IoU* metric, evaluating each one for the 14 classes; the closer the value is to 1, the better the classification:

(2) $$IoU=\frac{target\cap prediction}{target\cup prediction}.$$

### 4.3. Depth Estimation Qualitative Results

**Figure 11:**
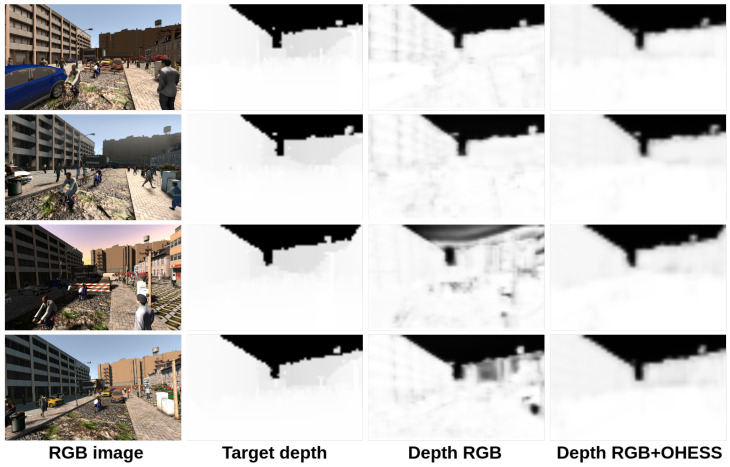
Results obtained with the SCRX CNN model on the 2016 SYNTHIA dataset.

Before showing the results obtained with the dataset proposed for this work, let us explain why the 2016 SYNTHIA dataset [33]—the first version of this dataset—has not been used. First, the previous version does not have a separation of the data in training and testing. Secondly, in the representation of depth, some depth values are very close, that is, some objects such as vehicles are lost in the background of the image; some results from these experiments performed with this dataset are shown in Figure 11.

We used the L2 Norm as the loss function in all the depth estimation architectures, given by Equation (3), where $y^{\prime }$ is the estimated depth map, *y* is the target depth map, and *n* is the total number of pixels per image. We decided to use this loss function because these CNN models perform regressions:

(3) $$L2=\frac{1}{2n}\sum_{i=1}^{n}{∥y\left(i\right)-y^{\prime }\left(i\right)∥}_{2}^{2}.$$

#### 4.3.1. SCRX Model Qualitative Results

**Figure 12:**
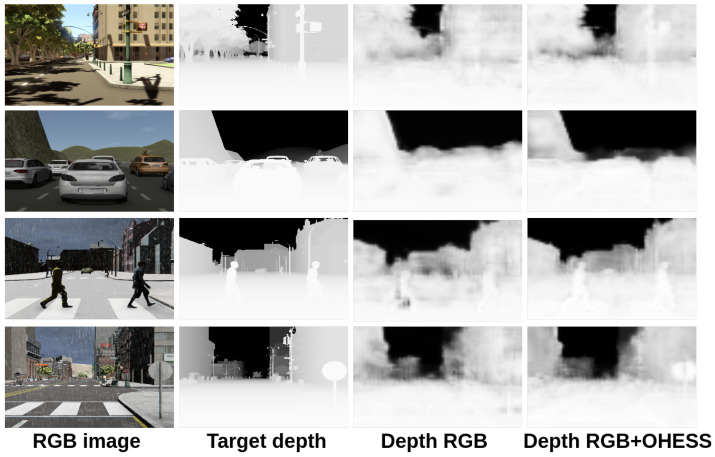
Results obtained with the SCRX CNN model.

**Figure 13:**
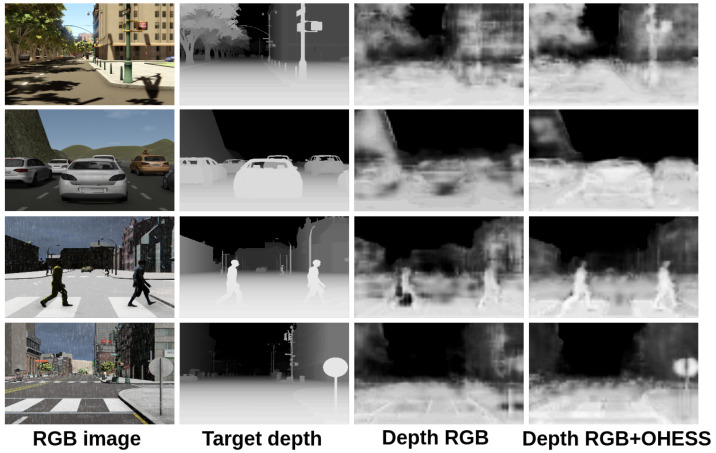
Results obtained with the SCRX CNN model (visually enhanced).

In Figure 12, the results obtained with and without the semantic segmentation are shown; the best results were obtained using the RGB image plus the given OHESS segmentation, improving results when only the RGB image is used for estimating depth. In Figure 13, the results are shown after a histogram image equalization, to improve contrast and allow better visualization.

#### 4.3.2. 3D CNNs Qualitative Results

**Figure 14:**
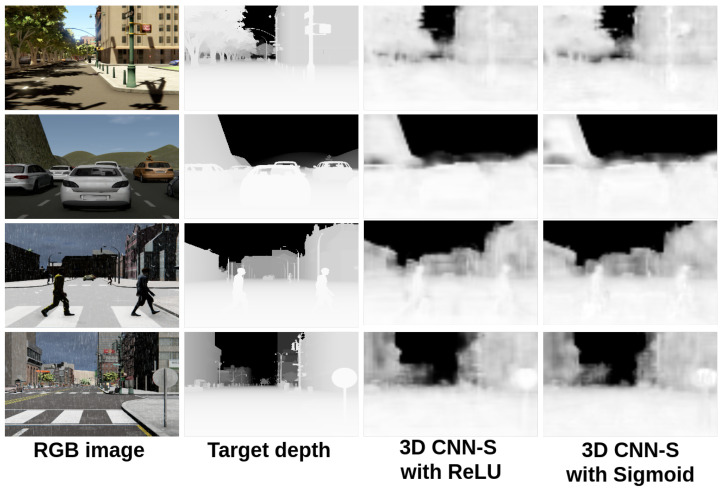
Results obtained with the 3D CNN-S model.

**Figure 15:**
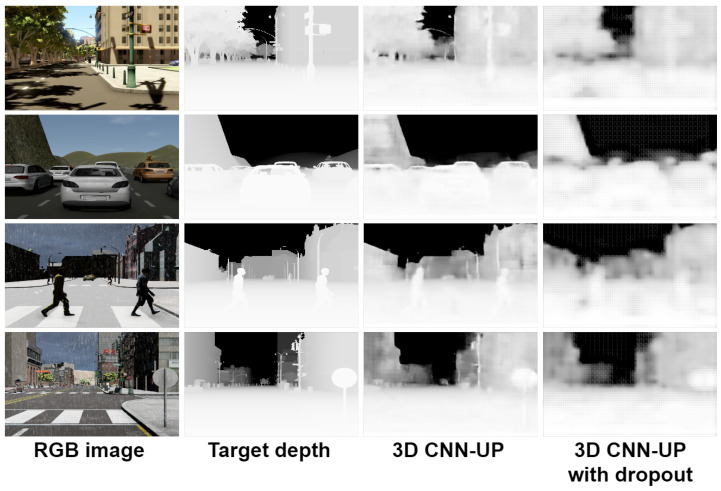
Results obtained with the 3D CNN-UP model.

In Figure 14 and Figure 15, we show a comparison between all the results obtained with the proposed 3D CNN models. To show the efficiency of the models in the estimation of the depth, they were trained using the given OHESS segmentation.

**Figure 16:**
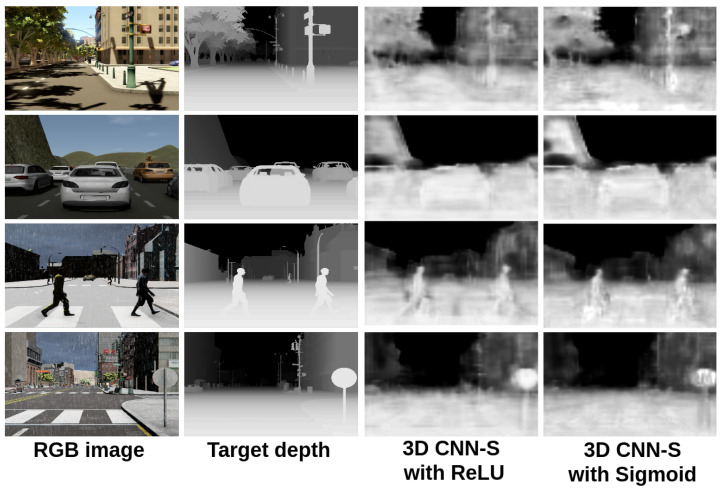
Results obtained with the 3D CNN-S model (visually enhanced).

**Figure 17:**
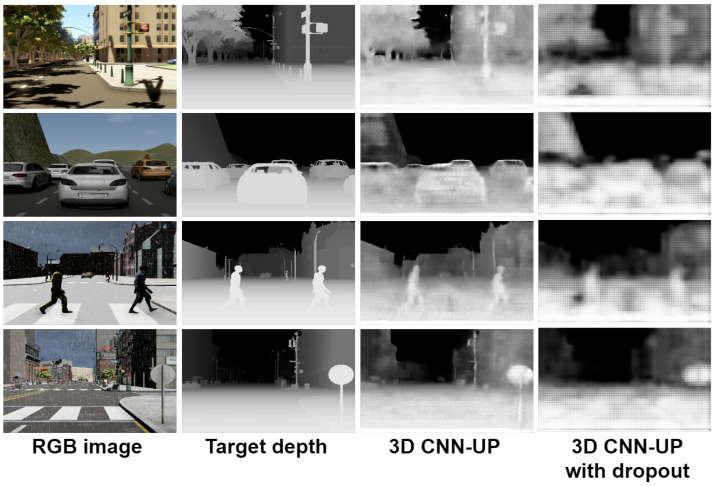
Results obtained with the 3D CNN-UP model (visually enhanced).

The best results were obtained using the 3D CNN-UP model without dropout, being better than those obtained using the 3D CNN-UP with dropout. In Figure 16 and Figure 17, the same results are shown after a histogram equalization, to improve contrast and allow better visualization.

### 4.4. Hybrid Semantic Segmentation and Depth Estimation: SSegDep-Net

**Figure 18:**
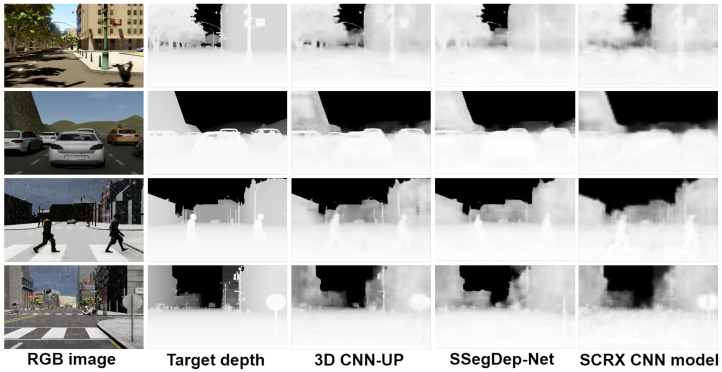
Comparison between the best results of all the CNN models.

**Figure 19:**
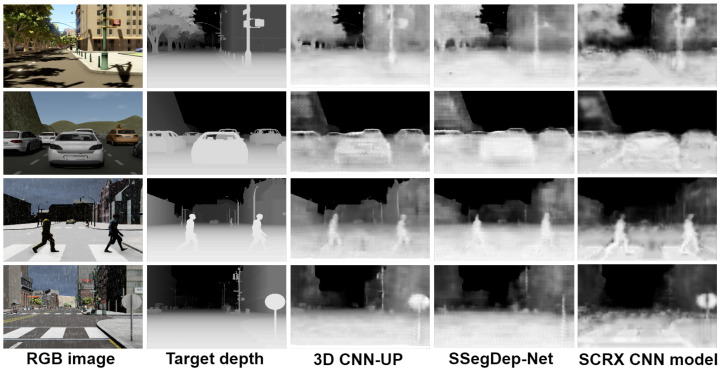
Comparison between the best results of all the CNN models (visually enhanced).

The SSegDep-Net model consists of two modules capable of obtaining semantic segmentation and depth estimation. According to the previous results, the depth estimation module is built by using the 3D CNN-UP model, and the semantic segmentation module is built by using the U-Net model. Once both modules are trained separately, we build the SSegDep-Net and perform the testing operation. The semantic segmentation module is obtained by using the U-Net model and the depth estimation module is the 3D CNN-UP; we chose this model because it yielded the best results when performing depth estimation. A comparison between the best results obtained in this paper is shown in Figure 18 and the visually-enhanced results are shown in Figure 19.

### 4.5. Implementation and Evaluation

To implement, train, and test our models (https://github.com/EduardoValdezRdz/Depth-Estimation-using-3d-2d-CNNs, accessed on 20 February 2022), we used the Python toolboxes, MxNet [34] and Keras [35]. All the experiments were run in two GPUs NVIDIA GTX 1080Ti. We trained separately the SCRX, the 3D CNN-S, and the 3D CNN-UP models with Back Propagation (BP) [36] and Stochastic Gradient Descent (SGD) [29] with a learning rate equal to 0.001. The U-Net model was trained using the Adam optimizer with a learning rate equal to 0.001. To evaluate the performance of all proposed models used as depth estimators, we selected state-of-the-art metrics (described in the Appendix A) that quantify per-pixel differences between the target depth map *y* and the estimated depth map $y^{\prime }$ [37]. Table 2, Table 3 and Table 4 show the quantitative results between all the proposed models. Unfortunately, there are no recent works to compare with using this version of the SYNTHIA-AL dataset. The closest work to compare with is proposed by Genovese et al. [19], because they use the same dataset used in this work, however they do not perform an analysis between their work and the state of the art papers, and they use different evaluation metrics. Nevertheless, according to Honauer [37], although the interpretation of results depends on the application, error metrics near 0 imply a good performance of the algorithm. In Table 5, we compare the training and test time for all our experiments. In general, the testing time is similar between all the experiments because once the CNNs were trained, we perform a forward propagation of the input images, taking approximately one second to perform the test.

**Figure 20:**
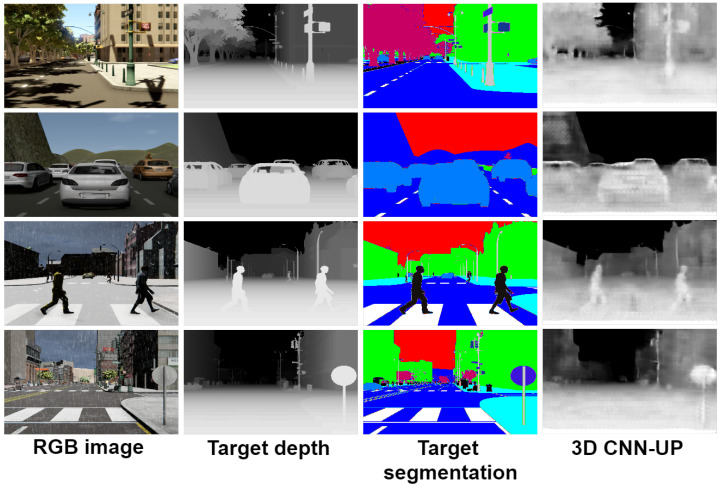
Results from the 3D CNN-UP visually enhanced; this model uses the segmentation given by the dataset.

**Figure 21:**
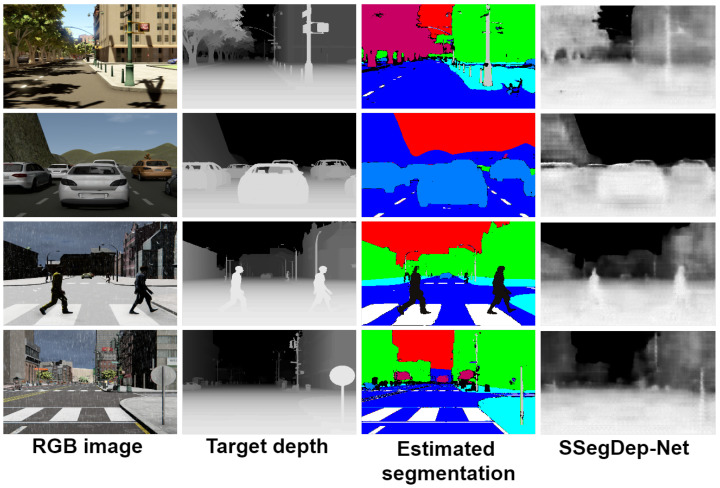
Results from the SSegDep-Net visually enhanced; this model uses the estimated segmentation given by the U-Net.

The proposed SSegDep-Net model had a comparable performance, although it showed lower results in qualitative metrics due to some errors found in the semantic segmentation module. Figure 20 shows results of the 3D CNN-UP model, where it can be observed how the target segmentation improves the results; compare this with Figure 21, where results of the SSegDep-Net are depicted—we can see some objects that could not be identified by the U-Net, and therefore were not considered in the depth estimation (see the last row of Figure 21).

**Discussion.** As observed in Table 2 and Table 3, the best results for the depth estimation were obtained using 3D CNNs, specifically using the 3D CNN-UP model with no dropout. On the other hand, the SCRX model using One-Hot Encoded Semantic Segmentation along with the RGB image showed good results too, despite that in the RMSL and RMSLSI metrics, it was outperformed by the RGB model without OHESS. We believe that this is since the 2D convolution flattens the entire input and does not consider the input as a whole, compared to the 3D convolution, which takes the entire input volume from 3D kernels, so that we can state that semantic segmentation is closely related to the RGB image’s information.

**Figure 22:**
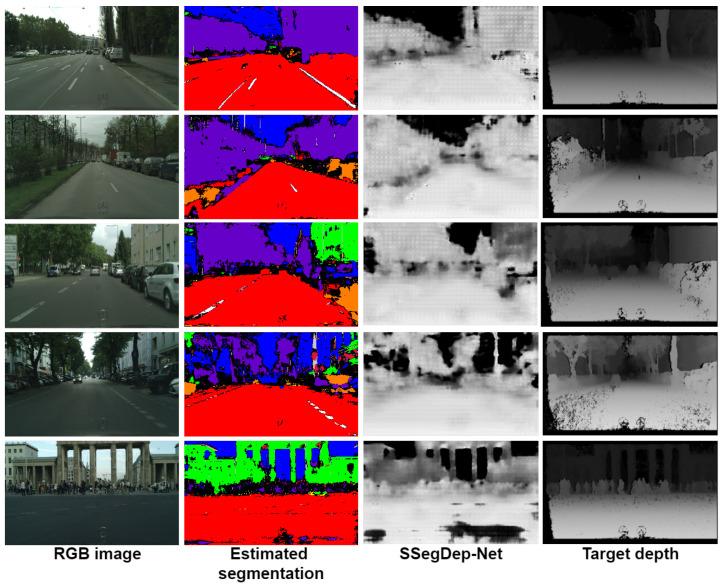
Results from the SSegDep-Net model on the Cityscapes dataset.

**Testing SSegDep-Net on real environments.** In this experiment, we observe some results using the Cityscapes dataset [32], although we mentioned that this dataset is not suitable for training because the training depth maps are incomplete and need preprocessing in order to improve their quality. We obtained some results to show the efficiency of our proposed model. In this experiment, we used the trained SSegDep-Net model by feeding only the RGB image to the model. In Figure 22, we show the results of our model with our proposed model, and from these results we can infer that our model depends on semantic segmentation because it fails to detect some small objects, such as vehicles and pedestrians, however for larger objects, such as the sky, buildings, and streets it shows good results. In Table 6 and Table 7, we compare our results with Wang work [38], which uses the Cityscapes dataset. We obtained similar results, however further analysis with other methodologies will be made in future works. In order to improve these results, in future work we propose to modify some hyperparameters or use a different semantic segmentation model because the depth estimation module has shown good results when the given segmentation is given by the dataset.

## 5. Conclusions and Future Work

In this work, we explored 2D, 3D CNN models, and particularly a hybrid 2D–3D CNN model capable of obtaining semantic segmentation and depth estimation at the same time. We found that helping the CNN models with additional information such as One-Hot Encoded Semantic Segmentation, aids in separating objects, and thus, obtaining a better depth estimation: Knowing the shape and position of objects in the scene, a CNN can estimate their depth distance with greater accuracy. Although we showed that local information is helpful for estimate depth in 2D CNN models, the best way to process all input planes is by using a 3D CNN model, due to the structure and operation volumes it creates when performing 3D convolutions. Tests were performed on a recent dataset, and therefore results of other methods on these datasets are not yet available. However, we found that using both 2D and 3D CNNs with additional information improves depth estimation from a single RGB image; we attested the importance of semantic segmentation in depth estimation, as it helps to locate the objects in the image. On the other hand, we also found some deficiencies in the U-Net model, which can be solved in future work by modifying some parameters during the training process. As future work, we also propose to analyze the effect of hyperparameters, such as the number of kernels or learning optimizers; additionally, the use of other semantic segmentation architectures will be explored in order to improve the semantic segmentation results. Another future task is to train both models using real data and synthetic data to show if adding additional classes may improve the results.

## Figures and Tables

**Table 1:** Semantic segmentation IoU (Intersection over Union) obtained for each class.

SYNTHIA-AL Dataset	
**Class**	**IoU**
Miscellaneous	0.9089
Sky	0.8954
Building	0.7780
Road	0.8193
Sidewalk	0.9251
Fence	0.8098
Vegetation	0.7521
Pole	0.6287
Vehicle	0.8415
Sign	0.6528
Pedestrian	0.8839
Cyclist	0.7570
Lanemark	0.9165
Traffic light	0.6878
Average	**0.8041**

**Table 2:** Quantitative results using the RMS, MQE, and RMSL error metrics (see the Appendix A. 3D CNN-S${}^{relu}$ uses ReLU in last layer, 3D CNN-S${}^{sigm}$ uses sigmoid in the last layer, and 3D CNN-UP${}^{do}$ adds dropout).

		Lower Is Better		
		**RMS**	**MQE**	**RMSL**
**SCRX** **Model**	**RGB**	0.1258	0.0245	**0.6252**
	**RGB+OHESS**	**0.0752**	**0.0068**	0.8457
**3D** **CNN** **Models**	**3D CNN-S${}^{relu}$**	0.0887	0.0101	1.2255
	**3D CNN-S${}^{sigm}$**	0.0885	0.0097	1.2176
	**3D CNN-UP**	**0.0676**	**0.0062**	**0.1042**
	**3D CNN-UP${}^{do}$**	0.1025	0.0135	0.5613
	**SSegDep-Net**	**0.0944**	**0.0126**	**0.1402**

**Table 3:** Quantitative results using the RMSLSI, ABSR, and ABSQ error metrics (see the Appendix A. 3D CNN-S${}^{relu}$ uses ReLU in the last layer, 3D CNN-S${}^{sigm}$ uses sigmoid in the last layer, and 3D CNN-UP${}^{do}$ adds dropout).

		Lower Is Better		
		**RMSLSI**	**ABSR**	**ABSQ**
**SCRX** **Model**	**RGB**	**0.3055**	0.2470	0.0463
	**RGB+OHESS**	0.4170	**0.2435**	**0.0282**
**3D** **CNN** **Models**	**3D CNN-S${}^{relu}$**	0.5799	0.2841	0.0612
	**3D CNN-S${}^{sigm}$**	0.5580	0.0054	0.0500
	**3D CNN-UP**	**0.0282**	**0.0054**	**0.0050**
	**3D CNN-UP${}^{do}$**	0.2730	0.2930	0.0790
	**SSegDep-Net**	**0.0467**	**0.0125**	**0.0087**

**Table 4:** Quantitative results using the threshold metric σ (see the Appendix A. 3D CNN-S${}^{relu}$ uses ReLU in the last layer, 3D CNN-S${}^{sigm}$ uses sigmoid in the last layer, and 3D CNN-UP${}^{do}$ adds dropout).

		Higher Is Better		
		** $\sigma_{1}$ **	** $\sigma_{2}$ **	** $\sigma_{3}$ **
**SCRX** **Model**	**RGB**	0.7573	0.7900	0.8021
	**RGB+OHESS**	**0.8021**	**0.8065**	**0.8102**
**3D** **CNN** **Models**	**3D CNN-S${}^{relu}$**	0.7662	0.7974	0.8072
	**3D CNN-S${}^{sigm}$**	0.7768	0.7825	0.8025
	**3D CNN-UP**	**0.8919**	**0.9105**	**0.9500**
	**3D CNN-UP${}^{do}$**	0.8454	0.8347	0.8433
	**SSegDep-Net**	**0.8610**	**0.8861**	**0.8929**

**Table 5:** Performance comparison for all experiments.

		Number of Iterations	Batch Size	Training Time (h)	Time to Test a Single Image (s)
**SCRX** **Model**	**RGB**	50	27	12	0.95
	**RGB+OHESS**	50	17	36	1.25
**3D CNN** **Models**	**3D CNN-S**	50	10	72	1.25
	**3D CNN-S**	50	10	72	1.55
	**3D CNN-UP**	50	10	72	0.87
	**3D CNN-UP**	50	10	72	0.98
**2D CNN** **Model**	**U-Net**	3	27	24	0.68
**Hybrid** **CNN Model**	**SSegDep-Net**	-	-	-	0.83

**Table 6:** Quantitative results using the RMS, MQE, RMSL, RMSLSI, ABSR, and ABSQ error metrics (see the Appendix A). Lower values are better.

	RMS	MQE	RMSL	RMSLI	ABSR	ABSQ
SSegDep-Net	0.1196	0.0225	0.01732	0.2356	**0.1638**	0.0129
SemiMTL [38]	**0.0755**	-	-	-	0.334	-

**Table 7:** Quantitative results using the threshold metric σ (see the Appendix A). Higher values are better.

	$\sigma_{1}$	$\sigma_{2}$	$\sigma_{3}$
SSegDep-Net	**0.8323**	**0.8422**	0.8587
SemiMTL [38]	0.6148	0.8300	**0.9190**

## Data Availability

The authors are committed to providing access to all the necessary information so that readers can fully reproduce the results presented in this work. Used datasets are publicly available.

## Appendix A. Evaluation Metrics

Root Mean Square Error:	$RMSE=\sqrt{\frac{1}{\left|T\right|}\sum_{y^{\prime }\epsilon \left|T\right|}{\left(y-y^{\prime }\right)}^{2}}$
Mean Quadratic Error:	$MQE=\frac{1}{\left|T\right|}\sum_{y^{\prime }\epsilon \left|T\right|}{\left(y-y^{\prime }\right)}^{2}$
Logarithmic Root Mean Square Error:	$RMSL=\sqrt{\frac{1}{\left|T\right|}\sum_{y^{\prime }\epsilon \left|T\right|}{\left(log\left(y\right)-log\left(y^{\prime }\right)\right)}^{2}}$
Logarithmic Root Mean Square Error Scale Invariant:	$RLSI=\frac{1}{\left|T\right|}\sum_{y^{\prime }\epsilon \left|T\right|}{\left(log\left(y\right)-log\left(y^{\prime }\right)\right)}^{2}$
Absolute Relative Difference:	$ABSR=\frac{1}{\left|T\right|}\sum_{y^{\prime }\epsilon \left|T\right|}\frac{\left|y-y^{\prime }\right|}{y^{\prime }}$
Squared Relative Difference:	$ABSQ=\frac{1}{\left|T\right|}\sum_{y^{\prime }\epsilon \left|T\right|}\frac{{∥y-y^{\prime }∥}^{2}}{y^{\prime }}$
Threshold ($\sigma_{1}$, $\sigma_{2}$, $\sigma_{3}$):	$\%\;of\;y\;such\;that\;max\left(\frac{y^{\prime }}{y},\frac{y}{y^{\prime }}\right)<\sigma_{i},$
	$where:\;\sigma_{i}=1.25^{i},i=1,2,3$
Reconstructed depth map	$y^{\prime }$
Target depth map	*y*
Number of pixels in the images	*T*
